# 多域整合治疗复杂原发性肺肉瘤样癌1例

**DOI:** 10.3779/j.issn.1009-3419.2024.102.04

**Published:** 2024-02-20

**Authors:** Xiaosen HUO, Hang ZOU, Yanyan DONG, Yuan LI, Lingjie BIAN, Lei LI, Hongwu WANG

**Affiliations:** 101121 北京，北京中医药大学东直门医院呼吸病中心; Respiratory Disease Center, Dongzhimen Hospital of Beijing University of Chinese Medicine, Beijing 101121, China

**Keywords:** 肺肉瘤样癌, 肺肿瘤, 中央气道狭窄, Pulmonary sarcomatoid carcinoma, Lung neoplasms, Central airway stenosis

## Abstract

肺肉瘤样癌（pulmonary sarcomatoid carcinoma, PSC）是一类罕见的、恶性程度较高的肺部肿瘤，分为多形性癌、梭形细胞癌、巨细胞癌、癌肉瘤和肺母细胞瘤5种病理类型。PSC发病隐匿，临床症状和体征无特异性，与肿瘤生长和侵袭部位密切相关，可表现为刺激性咳嗽、痰中带血丝、呼吸困难或胸痛等。PSC易早期出现转移，多数患者就诊时已是局部进展期或晚期，中位生存时间为9个月。我呼吸病中心诊治1例致中央气道狭窄90%的原发性PSC患者；通过血管介入和气管镜下介入联合治疗的方法拯救了患者生命，延长了生存时间，术后随访19个月，效果满意，现报道如下，以供临床借鉴。

## Abstract (en)

Pulmonary sarcomatoid carcinoma (PSC) is a rare and highly malignant tumor, which includes the following five pathologic types: pleomorphic carcinoma, spindle cell carcinoma, giant cell carcinoma, carcinosarcoma and pulmonary blastoma. The onset of PSC is occult with non-specific clinical symptoms and signs. The clinical manifestations include irritating cough, bloody sputum, dyspnea, chest pain and so on, which are closely related to the growth and invasion site of the tumor. PSC tends to metastasize early, so most patients are already in local advanced stage or advanced stage with a median survival of 9 months at the time of hospital visit. A patient with primary PSC which led to 90% stenosis in central airway was treated by combined method of vascular and tracheoscopic intervention in our respiratory center. This treatment prolonged the patient’s survival time and got a satisfactory effect at 19-month follow-up after surgery. Herein we report the case for clinical reference.

## 1 病例介绍

患者，男性，68岁，主因“间断咳嗽喘息2年余，加重4月余”于2022年4月4日就诊北京中医药大学东直门医院呼吸病中心。患者2020年3月中旬无明显诱因出现间断咳嗽及喘息，活动后加重，未引起重视；2021年12月初，患者咳喘加重，伴少量黄黏痰，偶有痰中带血丝，无发热，无胸痛，可平卧位休息，就诊天津某医院，诊断为：左主支气管占位性病变、左全肺不张、右侧肺气肿合并部分肺大疱。气管镜活检左主支气管肿物时，渗血明显，及时止血并中止手术，病理仅提示为坏死组织和少许纤维组织。抗炎、化痰及平喘保守治疗后，病情逐渐加重，且不能平卧，遂急诊收入我呼吸病中心。既往史：慢性阻塞性肺疾病和肺动脉高压（轻度）。个人史：吸烟史50年，60支/d，否认放射性物质及粉尘接触史。体格检查：心率103次/min，呼吸20次/min，血压115/70 mmHg，精神差，喘息貌，口唇紫绀，双侧胸廓不对称，左侧塌陷，右侧膨隆；听诊左肺未闻及呼吸音，右肺呼吸音粗；叩诊左侧实音，右侧过清音。卡氏体能状态（Karnofsky performance status, KPS）评分20分，气促评分4分。辅助检查：高流量吸氧6 L/min，血气分析：pH 7.434，动脉血二氧化碳分压35.7 mmHg，动脉血氧气分压105 mmHg，标准碱剩余-0.4%，氧饱和度98.6%，血浆碳酸氢盐浓度23.9 mmol/L，标准碳酸氢盐浓度24.4 mmol/L；血常规：白细胞11.78×10^9^/L，血红蛋白125 g/L，中性粒细胞百分率83.10%。临床诊断：中央气道狭窄（III区）^[1]^、中央气道肿物、左肺肿物、左全肺不张、I型呼吸衰竭、肺气肿、肺大疱和肺动脉高压。

治疗及转归：患者急诊入院后，立即进行全身麻醉，快速经口插入硬质支气管镜，侧孔连接高频通气并维持血氧饱和度，支气管镜经其后孔进行消瘤操作。支气管镜下见管内型^[2]^肿物阻塞中央气道III区管腔约90%（图1A）和完全阻塞左主支气管VII及VIII区管腔（图1B）。采用圈套器、氩气刀和冷冻等结合方法消除肿瘤，显露出隆突（图1C）和左主支气管管腔（图1D）。充分灌洗并吸引支气管内脓性分泌物，见左上叶支气管开口被管内型和管壁型肿物完全阻塞（图1E），左下叶各段管腔通畅（图1F）。消瘤过程中，渗血约200 mL，被迫中止手术。术后病理（图2A、2B）：肉瘤样癌，具有鳞状上皮细胞和横纹肌肉瘤分化。免疫组化染色结果：抗细胞角蛋白1/3（anti-cytokeratin 1/3, AE1/3）（-）、波形蛋白（Vimentin, VIM）（+）、上皮细胞黏附分子（epithelial cell adhesion molecule, EMA）（-）、P53（少数+）、P63（+）、P40（+）、分化簇34（cluster designation 34, CD34）（脉管+）、CD56（部分+）、Sal-like蛋白4（Sal-like protein 4, SALL4）（-）、平滑肌激动蛋白（smooth muscle actin, SMA）（-）、肌细胞生成素（myogenin, MyoG）（-）、低分子细胞角蛋白（low molecule cytokeratin, CAM5.2）（-）、细胞角蛋白7（cytokeratin-7, CK7）（-）、CK5/6（部分+）、转录因子2免疫球蛋白（sex-determining region-Y homeobox-2, SOX2）（+）、嗜铬粒蛋白A（chromogranin A, CgA）（-）、突触核蛋白（synuclein, Syn）（-）、肾小球足突细胞膜黏蛋白（podoplanin, D2-40）（个别+）和甲状腺转录因子1（transcription termination factor 1, TTF-1）（-）。未发现基因突变和程序性死亡配体1（programmed cell death ligand 1, PD-L1）表达。术后当天，患者出现左侧气胸，给予胸腔闭式引流处理。复查胸部增强计算机断层扫描（computed tomography, CT）：左侧胸腔积气，左上叶肿物明显强化及上叶不张，左下叶复张（图3A、3B）。经抗炎、化痰及持续胸腔引流后，左侧胸腔积气完全排出，左上叶仍然不张，左下叶完全复张（图3C）。2022年4月18日，在数字减影血管造影（digital subtraction angiography, DSA）下，Seldinger法穿刺右股动脉、置入5 F导管鞘，造影显示左侧支气管动脉供血左上叶肿物，呈网状显影（图3D），之后进行支气管动脉灌注化疗及栓塞治疗。将顺铂30 mg溶于50 mL生理盐水，缓慢推注20 min，然后350-560 µm明胶海绵栓塞供血动脉，直至左上叶肿物网状显影消失。2022年4月20日，全身麻醉后支气管镜下继续消除左肺上叶各段管腔内肿物，顺铂10 mg溶于20 mL生理盐水并注射于管壁肿物基底部。术后左肺上叶前段、舌段及下叶完全复张（图3E）。复查动脉血气数值在正常范围，KPS评分70分，气促评分2分。确定诊断：（1）左肺恶性肿瘤（原发性中央型左肺肉瘤样癌cT4N0M0，IIIA期）；（2）中央气道侵犯伴重度狭窄（VII和VIII区）；（3）左肺不张；（4）I型呼吸衰竭；（5）肺气肿；（6）肺大疱；（7）肺动脉高压。此后，患者2次出现左主支气管、左上叶支气管前段和舌段管内型肿物生长导致左上叶不张，分别于2022年7月5日和10月24日行上述血管介入和气管镜介入联合治疗，术后左上叶前段、舌段和左下叶均可复张。因新冠疫情影响，2023年3月24日患者第4次随诊，左上叶支气管管腔肿物无法消除再通，左上叶未能复张。2023年9月20日患者第5次随诊，左主支气管肿物也呈管内型和管壁型混合生长，未能消瘤再通管腔，致使左肺完全不张（图3F）。电话随访，患者于2023年11月初因呼吸衰竭死亡，生存时间19个月。

**图1:**
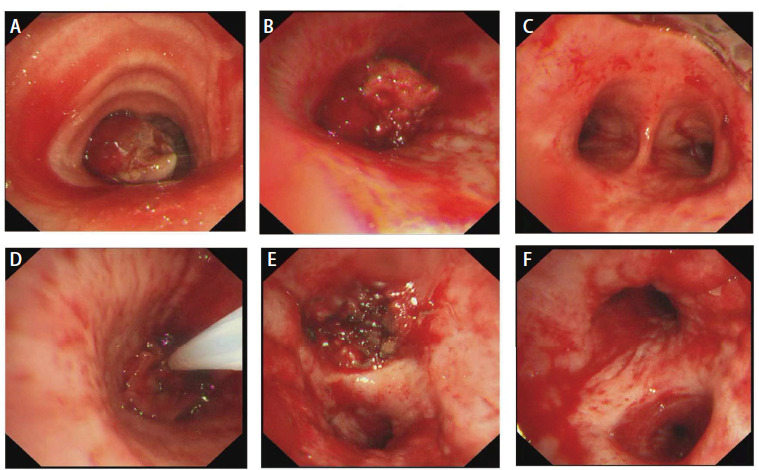
患者气管镜下治疗情况。A：中央气道肿物（III区）；B：左主支气管肿物（VII和VIII区）；C：中央气道肿物消除后，隆突显现；D：左主支气管肿物消除；E：左上叶支气管开口肿物；F：左下叶各段管腔通畅。

**图2:**
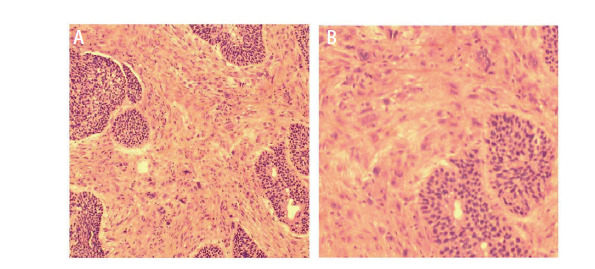
肿瘤HE染色结果（A：×100；B：×200）

**图3:**
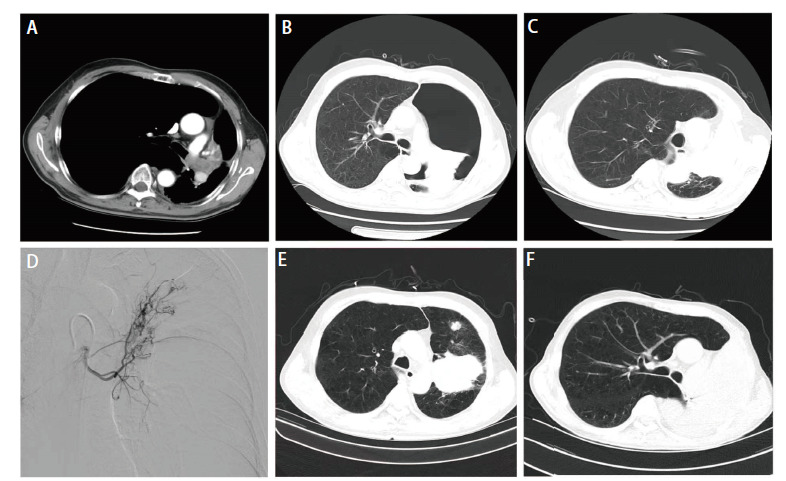
患者影像学资料。A：肿物强化；B：术后左侧气胸，左上叶不张；C：左下叶完全复张；D：左上叶肿物显影；E：肿物位于尖后段；F：左全肺不张。

## 2 讨论

PSC是含有肉瘤样成分的低分化非小细胞肺癌（non-small cell lung cancer, NSCLC），占原发肺部恶性肿瘤的0.3%-3%和NSCLC的1%^[3]^，由上皮癌组织通过上皮-间充质转化（epithelial-mesenchymal transition, EMT）而逐渐形成，好发于老年男性，平均年龄（63.3±9.8）岁，与吸烟史有关^[4]^。目前，早期手术治疗仍是最有效的治疗方案^[4,5]^，但术后中位生存时间为20个月^[6]^，5年生存率为3.85%，劣于NSCLC中腺癌和鳞癌的预后^[4]^。对于肿瘤导致中央气道重度狭窄的患者来说，应先开通气道保证生命、改善生存质量，后期再根据病理及基因突变的情况，采用化疗、靶向治疗或免疫治疗等维持治疗^[7,8]^。低氧血症增加了麻醉风险，维持术中血氧饱和度是顺利手术的关键。硬质气管镜与高频通气结合能够很好地维持血氧饱和度，并通过其后孔顺利进行手术操作。该患者第1次就诊时，中央气道III、VII、VIII区和左上叶部分肺段内肿物属于管内型。根据王洪武教授的经验^[9]^，通过套取、冻取及氩气刀等方法可完全取出管内型肿物，阻塞的肺不张可完全缓解；而管壁型肿物具有沿管壁长轴浸润生长的特点，致使管壁全层、全周或近全周增厚，因此气管/支气管腔内呈现混合型肿物生长时，完全清除肿物的可能性较小。患者术后当天出现了气胸，也说明了管壁型肿物破坏了正常的气管管壁结构，消瘤操作易穿透管壁。对于腔内混合型肿物应部分消除，必要时要结合内支架置入^[9]^。根据PSC的恶性特点以及该患者的随访过程，我们认为2个月入院随访是适宜的，能够及时消除气道内肿物，保证正常肺组织复张。另外，PSC还具有富血型肿瘤特点，尤其是中央型肿瘤，术中出血较多，因此消瘤前的支气管动脉栓塞是必要的。

目前，PSC对照研究少，各类报道结论^[4,10⇓-12]^存在差异，放化疗的治疗价值尚无定论；同时结合患者术后KPS评估，我们未进行放疗及全身静脉化疗。局部灌注化疗药物浓度可以达到全身静脉给药至局部浓度的数倍，增强药物的细胞毒性作用。所以，我们采用了支气管动脉灌注化疗和气管镜下肿瘤局部注射治疗的方式。尽管文献^[13⇓-15]^报道，PSC中有较高的基因突变率和PD-L1表达，但该患者基因检测未发现突变和PD-L1表达，可能与其病理亚型有关。因此，有效的放化疗方案、靶向治疗及免疫治疗仍需要进行更多的临床研究。

对于生存期较短或无法预估生存期的晚期肺癌患者来说，王洪武教授^[8]^提出的晚期肺癌多域整合治疗策略为提高肿瘤诊疗效能提供了思路。通过血管介入（海）和气管镜下介入（陆）的联合应用不仅挽救了患者生命、提高生存质量，而且延长了生存时间并赢得了后续治疗的宝贵时间。因此，多域整合治疗策略具有高效和创新的特点，值得在临床中推广和借鉴。

**Competing interests**

The authors declare that they have no competing interests.
